# The emerging role of exosomes in innate immunity, diagnosis and therapy

**DOI:** 10.3389/fimmu.2022.1085057

**Published:** 2023-01-16

**Authors:** Prakash Gangadaran, Harishkumar Madhyastha, Radha Madhyastha, Ramya Lakshmi Rajendran, Yuichi Nakajima, Nozomi Watanabe, Anoop Kumar G. Velikkakath, Chae Moon Hong, Rahul Velikkakath Gopi, Gothandam Kodiveri Muthukalianan, Abilash Valsala Gopalakrishnan, Madhan Jeyaraman, Byeong-Cheol Ahn

**Affiliations:** ^1^ BK21 FOUR KNU Convergence Educational Program of Biomedical Sciences for Creative Future Talents, Department of Biomedical Science, School of Medicine, Kyungpook National University, Daegu, Republic of Korea; ^2^ Department of Nuclear Medicine, School of Medicine, Kyungpook National University, Kyungpook National University Hospital, Daegu, Republic of Korea; ^3^ Department of Cardiovascular Physiology, Faculty of Medicine, University of Miyazaki, Miyazaki, Japan; ^4^ Center for System Biology and Molecular Medicine, Yenepoya Research center, Yenepoya (Deemed to be University), Mangaluru, Karnataka, India; ^5^ Department of Tissue Engineering and Regeneration Technologies, Sree Chitra Thirunal Institute of Medical Sciences and Technology, Thiruvananthapuram, India; ^6^ Department of Biomedical Sciences, Vellore Institute of Technology (VIT), Vellore, India; ^7^ Department of Orthopaedics, Faculty of Medicine, Sri Lalithambigai Medical College and Hospital, Dr MGR Educational and Research Institute, Chennai, Tamil Nadu, India

**Keywords:** exosomes, secretory cells, tissue inflammation, circulation, therapy

## Abstract

Exosomes, which are nano-sized transport bio-vehicles, play a pivotal role in maintaining homeostasis by exchanging genetic or metabolic information between different cells. Exosomes can also play a vital role in transferring virulent factors between the host and parasite, thereby regulating host gene expression and the immune interphase. The association of inflammation with disease development and the potential of exosomes to enhance or mitigate inflammatory pathways support the notion that exosomes have the potential to alter the course of a disease. Clinical trials exploring the role of exosomes in cancer, osteoporosis, and renal, neurological, and pulmonary disorders are currently underway. Notably, the information available on the signatory efficacy of exosomes in immune-related disorders remains elusive and sporadic. In this review, we discuss immune cell-derived exosomes and their application in immunotherapy, including those against autoimmune connective tissue diseases. Further, we have elucidated our views on the major issues in immune-related pathophysiological processes. Therefore, the information presented in this review highlights the role of exosomes as promising strategies and clinical tools for immune regulation.

## 1 Introduction

Extracellular vesicles encompass a large heterogeneous family of membrane-bound structures, such as microvesicles, exosomes, and apoptotic bodies. The Heine group initially named all vesicles derived from plasma membrane exosomes (1). Earlier studies by the Turbide group reported that reticulocyte culture releases exosomal vesicles that can be harvested by ultracentrifugation. Further analysis of these vesicles revealed the presence of transferrin receptors, nucleoside transporters, glucose transporters, acetylcholinesterase, and Na^+^-independent amino acid transporter (2). Exosomes are capable of transferring miRNAs and mRNAs between cells (3). Almost all cell types release exosomes with varying molecular compositions and biogenesis pathways. Several bodily fluids, such as saliva, urine, breast milk, amniotic fluid, synovial fluid, serum, and ascites fluid, contain exosomes under physiological and pathological conditions. Exosomes were previously considered cellular waste generated due to cell damage and homeostasis. However, they have been extensively investigated after revealing the antitumor potential of exosomes carrying MHC-I and MHC-II (4).

Exosomes range from 30–150 nm in size (5, 6). Later research ascertained their genesis to be from the inward budding of endosomes, producing multivesicular bodies with intraluminal vesicles. Subsequently, proteins are incorporated into the invaginating membrane, and cytosolic components are engulfed and enclosed within intraluminal vesicles. The contents of exosomes can differ depending on the source and physiological conditions of the cells that release them (7). The fusion of multivesicular bodies with the plasma membrane results in the release of intraluminal vesicles into the extracellular space, giving rise to “exosomes” (2, 8, 9). This is primarily an endosomal sorting complex required for transport (ESCRT)-dependent processes. However, it reportedly functions independently of ESCRT complexes (10, 11). Alternatively, they may also be trafficked to the lysosomes for degradation. These exosomes are mini versions of parent cells and have emerged as critical mediators in inter-cell communication, functioning as vehicles delivering a complex cargo of proteins, lipids, and nucleic acids from the parent cells to other distant cells. Upon reaching recipient cells, they interact either *via* surface ligands or through the delivery of activated receptors or epigenetic modulation of the recipient cells *via* the cargo of bioactive cells, consequently modulating the physiology of recipient cells. Thus, exosomes play distinct roles in a multitude of physiological processes, such as immune response, cell differentiation, signal transduction, and antigen presentation. Various cells secrete exosomes; the cargo is unique to its cellular origin and thus can be used as a biomarker. Their ability to package biomolecules within them has facilitated their use as drug delivery systems (12–15).

**Figure 1:**
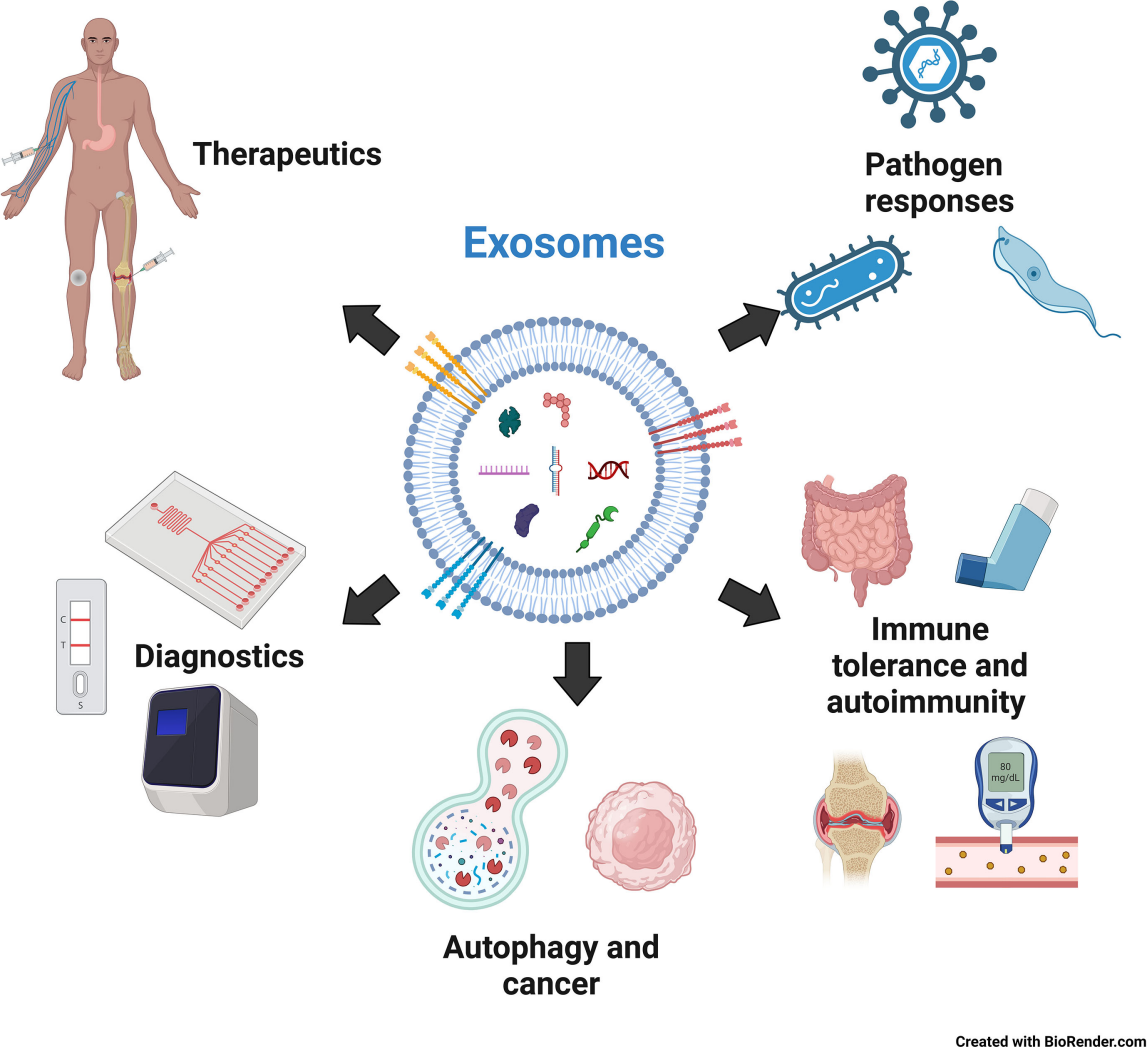
The role of exosomes in innate immunity, diagnosis and therapy. Created with BioRender.com.

This review focuses on one of the major physiological roles of exosomes: innate immune regulation. To maintain body function and homeostasis, protection against foreign agents is essential. This function is effectively performed by the immune system, a complex network of cells and organs that protects the organism against infectious agents, such as bacteria, viruses, and other pathogens. This is achieved through a combination of innate and adaptive immunity. Innate immunity is an antigen-non-specific, evolutionarily conserved system in most multicellular organisms, whereas lymphocytes mediate adaptive immunity through antigen specificity and memory. The innate immune system forms the first line of defense and comprises a network of cells, including monocytes/macrophages, dendritic cells, neutrophils, and natural killer cells, facilitating the earliest interactions between the host and pathogens. Upon entry of a foreign object, pattern-recognition receptors on immune cells, such as Toll-like receptors (TLRs), RIG-I-like receptors, and certain DNA sensors, such as cGAS, recognize various molecular signatures of invaders called pathogen-associated molecular patterns (PAMPs), which include numerous molecules, such as lipopolysaccharide (LPS) from gram-negative bacteria, peptidoglycans from gram-positive bacteria, and unmethylated CpG DNA from bacteria and viruses. Upon recognition of the entry of an invader, cell-cell communication is critical for swiftly spreading the message of infection and enabling the innate immune system to mount a broad response against the pathogen. Until recently, cytokines and chemokines have been extensively studied for their role as messengers in innate immunity. However, recent research has revealed that exosomes are also vital in this communication (16). This review mainly focuses on the mechanisms by which exosomes mediate the innate immune response of the host to pathogens (viruses, bacteria, and parasites) as well as act against their cells *via* autoimmunity. We also focus on using exosomes as diagnostic biomarkers and therapeutic agents. We also focus on using exosomes as diagnostic biomarkers and therapeutic agents (**Figure 1**).

## 2 Exosomes in pathogen response

Exosomes are released from pathogens and host cells. They modulate stimulatory or suppressive effects on the innate immune system through exosome-mediated intercellular communications. They are crucial in immune regulation, including antigen presentation, immune activation, immune suppression, and immune tolerance. Their immune activator or suppressor role depends mainly on the source of the exosomes and their biomolecular content. Exosomes derived from healthy human plasma samples contain various RNA species, such as mRNAs and noncoding regulatory RNAs, within these circulating vesicles (17–19). Pathways related to NF-κB activation and TLR cascades differ between exosomal mRNAs from naïve cells compared with those from LPS-stimulated cells, indicating significant changes in adaptive and innate immune processes. Exosomes are also carriers of critical soluble mediators such as cytokines. After LPS stimulation, RAW 264.7 mouse macrophages exhibited increased levels of cytokines, predominantly chemokines. Ten of the 16 cytokines secreted by LPS-stimulated RAW 264.7 cells were from cell-derived exosomes (20).

### 2.1 Exosomes in viral infections

Mounting evidence from several viral infections suggest that exosomes can transfer viral components, including proteins, genomic molecules, and receptors, from infected to healthy cells, thus promoting infection and inflammation. The delivery of viral receptors to target cells by exosomes renders the cells susceptible to viral entry.

Nef, the HIV protein, is enclosed within exosomes (21, 22); they are activated during the uptake of these exosomes by latent cells, rendering them susceptible to HIV infection (23). Nef also inhibits the generation of CD4+ exosomes from T cells and induces the death of CD4+ T cells. Consequently, immune cells suppress viral recognition (24–26). Mack et al. demonstrated that the transport of viral receptors such as chemokine receptor 5 (CCR5), the main co-receptor for HIV infection, from peripheral blood mononuclear and CCR5+ ovary cells to CCR5-null cells enhances HIV-1 infection (27). Moreover, exosomes produced by megakaryocytes and platelets harbor the HIV co-receptor C-X-C chemokine receptor type 4 (CXCR4) and increase the susceptibility of CXCR4-null cells to X4-HIV infection (28). Exosomes also promote infection by delivering viral nucleic acids to the uninfected cells. Exosomes from HIV-infected cells reportedly transmit transactivation response elements (29, 30). The transactivation response element at the 5′ ends of the HIV transcript copies and interacts with the Tat protein to produce viral RNAs, consequently generating miRNAs. These miRNAs can inhibit a Bcl-2-interacting protein, which ultimately promotes resistance to apoptosis and promotes virus production (29, 30). Conversely, exosomes from infected cells can suppress viral infection. For instance, human cytidine deaminase apolipoprotein B mRNA editing enzyme, catalytic subunit 3G (APOBEC3G) molecules present in exosomes from infected cells cause the deamination of cytosine residues to uracil in the minus strand of viral DNA during reverse transcription. Consequently, the viral infectivity factor of HIV type-1, which is essential for efficient viral replication, is not sorted into exosomes (31). Additionally, exosomes produced by infected cells contain cyclic guanosine monophosphate–adenosine monophosphate (cGAMP), which can trigger an antiviral response *via* innate immune responses and interferon upregulation (32, 33). HIV-related miRNAs, such as miRNA-88 and miRNA-99, induce endosomal nuclear factor-kappa B (NF-κB) and toll-like receptor 8 (TLR8) signaling, which activate the immune response against HIV *via* tumor necrosis factor-alpha (TNF-α) production from macrophages (34).

Although many viruses evade the pathogen-sensing pathway, the immune system adopts alternative pathogen-sensing strategies that are not challenged by viral evasion mechanisms. For instance, hepatitis C virus-permissive cells can selectively pack immunostimulatory viral RNA into exosomes and deliver them to neighboring plasmacytoid dendritic cells, triggering an antiviral IFN response *in vitro* (35).

**Figure 2:**
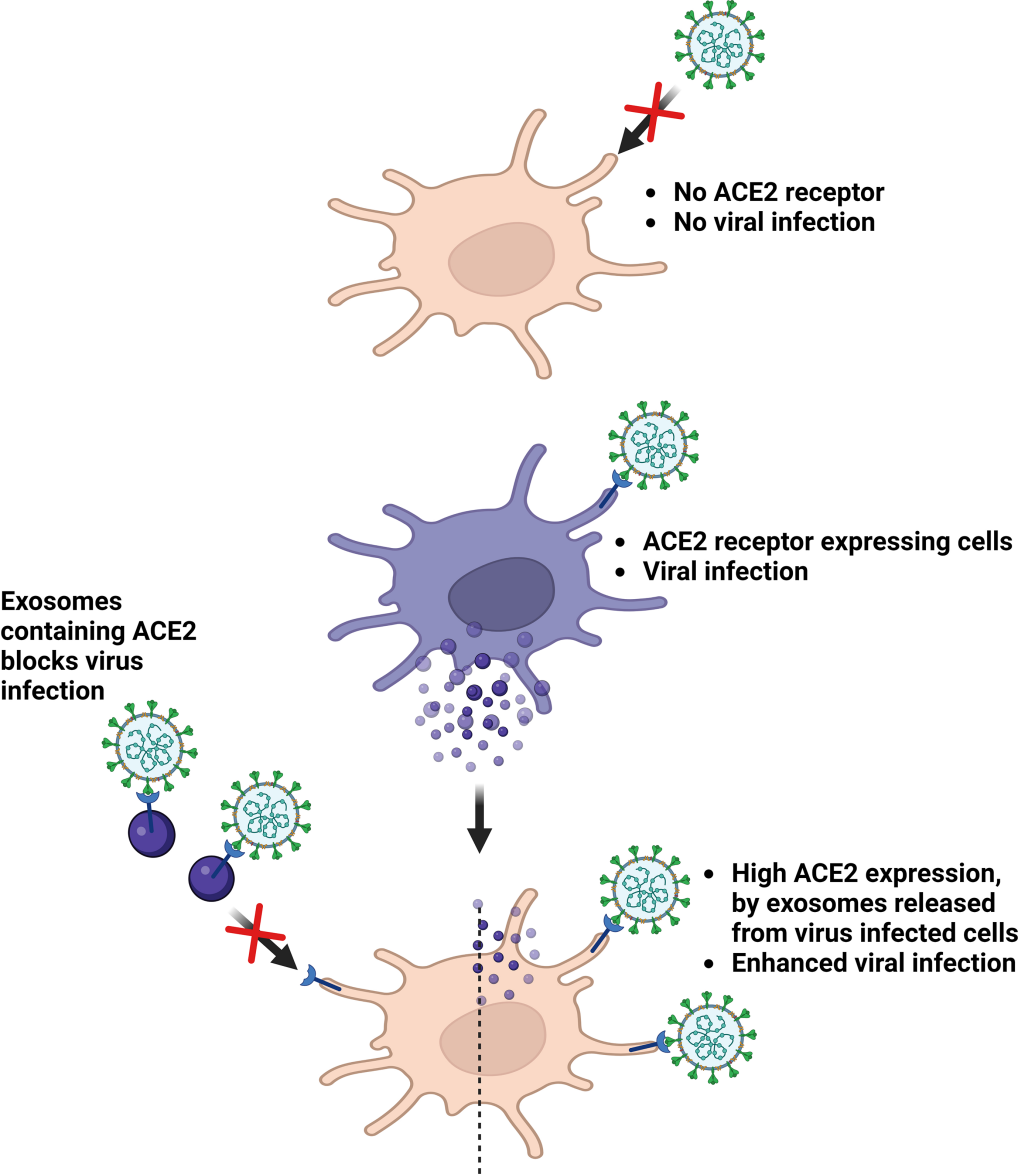
Roles of exosomes in virus infection. ACE2, Angiotensin converting enzyme 2. Created with BioRender.com.

Although knowledge of host-pathogen interactions in novel coronavirus SARS-CoV-2 infection remains limited, growing evidence suggest the role of exosomes in this process. In an *in vitro* study wherein, SARS-CoV was cultured in alveolar epithelial type II cells, viral particles were observed within double-membrane vesicles (36, 37). This immune system evasion could be attributed to the reinfection observed in several discharged and fully recovered patients. Exosomes can also transfer angiotensin-converting enzyme 2 (ACE2), the main viral receptor, to recipient cells (38), thereby rendering them susceptible to virus docking. Non-peer-reviewed observations from El-Shennawy et al. reported that exosomal ACE2 competes with cellular ACE2 to neutralize SARS-CoV-2 infection. The ACE2-expressing (ACE2+) exosomes blocked the binding of viral spike (S) protein RBD to ACE2+ cells in a dose-dependent manner. Notably, this was 400–700-fold more effective than that of vesicle-free recombinant human ACE2 extracellular domain protein (rhACE2). They could also prove that ACE2-containing exosomes could prevent SARS-CoV-2 pseudotype virus attachment and could infect human host cells at an efficacy 50–150-fold higher than that of rhACE2. Thus, ACE2+ exosomes serve as competitive inhibitors to block SARS-CoV-2 infection, suggesting a promising therapeutic role (39). The contribution of exosomes in COVID-19 infection has already been proven to be quite significant, and further research is essential to elucidate the exact mechanisms (**Figure 2** and **Table 1**).

**Table 1:** Exosomes in pathogen response.

Infections	Sources	Major contents	Effects	References
**Viral**	CD4+ T cells	Nef, the HIV protein	inhibits the generation of CD4+ exosomes, induces the death of CD4+ T cells	(21, 22, 24–26)
	megakaryocytes and platelets	C-X-C chemokine receptor type 4 (CXCR4)	increase the susceptibility of 15 CXCR4-null cells to X4-HIV infection	(28)
	HIV-infected cells	viral nucleic acids	promote infection by delivering viral 16 nucleic acids to the uninfected cells	(29, 30)
	HIV-infected cells	apolipoprotein B mRNA editing enzyme, catalytic subunit 3G	suppress viral infection	(31)
	HIV-infected cells	cyclic guanosine monophosphate–4 adenosine monophosphate (cGAMP)	trigger an antiviral response *via* innate immune 5 responses and interferon upregulation	(32, 33)
	macrophages	miRNA-88 and 6 miRNA-99	activate the immune response against HIV *via* tumor necrosis factor-alpha	(34)
	SARS-CoV was cultured in alveolar epithelial type II cells,	viral particles	immune system evasion	(36, 37)
	SARS-CoV-infected Endothelial Progenitor Cells	angiotensin-converting enzyme 2 (ACE2)	susceptible to virus docking	(38)
	SARS-CoV-infected cells	ACE2	blocked the binding of viral spike (S) protein RBD to ACE2+ cells	(39)
**Bacterial**	*Staphylococcus aureus*	bacterial pore-forming molecule, α-toxin	promote additional infection	(40)
	*Bacillus anthracis*-infected cells	lethal anthrax toxin	long-term and exosome-mediated long-distance infection	(41)
	methicillin-resistant *Staphylococcus aureus*	A disintegrin 16 and metalloproteinase domain-containing protein 10 (ADAM10)	bind the toxin to improve host survival	(42)
**Protozoan**	*Plasmodium falciparum*-infected red blood cells	Unknown	communicate with parasites and also promotes differentiation into sexual forms	(43)
	*Plasmodium yoelii*-infected reticulocytes	parasite protein	induce antigen presentation	(44)
	*Leishmania donovani*	Unknown	induce interleukin (IL)-8 secretion from macrophages	(45)
	*Trypanosoma cruzi*	Unknown	immune modulation and virulence	(46)
	*Leishmania mexicana-*treated macrophages	Unknown	upregulation of adenosine receptor 2a (Adora2a) on macrophages	(46, 47)
	*Trypanosoma bruceirhodesiense*	serum resistance-associated proteins	to evade innate immunity	(48)
	Culturing the intestinal epithelial cells with *Cryptosporidium parvum*	synaptosome-associated protein 23 (SNAP23)-	activates TLR4 signaling, leading to an upregulation of exosome secretion	(48)

### 2.2 Exosomes in bacterial infections

Exosomes can promote further infection by delivering bacterial molecules involved in pathogenesis. For instance, *Staphylococcus aureus*-derived exosomes harbor the bacterial pore-forming molecule α-toxin (40), and exosomes from *Bacillus anthracis*-infected cells have been observed to transport the lethal toxin virulence factor to sites distal to the infection (41). The work described (41, 42) sheds new light on how exosomes protect host cells by functioning as cellular decoys. The autophagy protein (ATG16L1) is required for protection against *Staphylococcus* aureus, which expresses α-toxin. This pore-forming toxin binds to the metalloprotease, a disintegrin and metalloproteinase domain-containing protein 10 (ADAM10), on the surface of various target cells and tissues. This study confirmed that ATG16L1 and other ATG proteins mediate protection against α-toxin by releasing ADAM10 on exosomes, which act as scavengers that can bind the toxin and improve host survival (**Table 1**).

### 2.3 Exosomes in protozoan infections

Several protozoan parasites, such as Plasmodium sp., Leishmania sp., and Trypanosoma sp., release exosomes. Malarial disease severity correlates with the production of exosomes from Plasmodium-infected cells (49). *Plasmodium falciparum*-infected red blood cells communicate with parasites using exosome-like vesicles. This also promotes differentiation into sexual forms (43). In *Plasmodium yoelii*-infected mice, parasite protein-containing exosomes are released from infected reticulocytes and can induce antigen presentation (44).

*Leishmania donovani* (promastigote and amastigote forms) and *Leishmania* primarily release exosomes that can induce interleukin (IL)-8 secretion from macrophages (45). Subsequently, neutrophils are recruited, and *Leishmania* can invade these cells and gain access to macrophages during the phagocytosis of infected neutrophils (45, 50). Metacyclic trypomastigote and non-infective (epimastigote) forms of *Trypanosoma cruzi* parasites release exosomes (46) that contain proteins associated with immune modulation and virulence. Leishmania spp. and *Trypanosoma cruzi* can also induce exosome release from the infected cells. Studies on *Leishmania mexicana*-treated macrophages *in vitro* suggested that exosomes released from infected cells can induce phosphorylation of signaling proteins and significantly upregulate immune-related genes, including adenosine receptor 2a (*Adora2a*) on macrophages (46, 47). *Trypanosoma brucei rhodesiense*-derived exosomes can transfer serum resistance-associated proteins to *Trypanosoma brucei*. The parasite requires serum resistance-associated proteins to circumvent the action of host lytic factors, thereby conferring the ability to evade innate immunity (48).

Culturing the intestinal epithelial cells with *Cryptosporidium parvum* activates TLR4 signaling, leading to an upregulation of exosome secretion from these cells. This process is mediated by synaptosome-associated protein 23 (SNAP23)-associated vesicular exocytosis. These released exosomes contain epithelial antimicrobial peptides that can bind to and decrease the viability and infectivity of *C. parvum* sporozoites (51), (**Table 1**).

## 3 Exosomes in immune tolerance and autoimmunity

The immune system is vital for the protection of the body and the maintenance of homeostasis. However, this system must be tightly regulated for the normal physiological functioning of the body to avoid response to non-threatening and beneficial antigens (for instance, microbiota), to distinguish self from non-self, and from over-reactivity to antigens (allergy or hyper-immunity). Despite stringent control, the immune system can sometimes react to its cells, tissues, and other components, contributing to autoimmunity. Exosomes also engage in these processes, which are detailed in the subsequent sections. Common auto immune thyroid diseases (AITDs) like Hashimoto’s thyroiditis and Graves’ diseases are class of immune disorders caused by the irregular infiltrations of lymphocytes and over productions of autoantibodies to cause the hypothyroidism or hyperthyroidism (52) and genetic along with environmental factors were acknowledged for contributing to development of AITDs (53, 54). Irregular activation/production of autoantibodies like thyroid peroxidase antibodies (TPOAb), thyroglobulin antibodies (TGAb) and thyrotropin receptor antibodies (TRAb) cause thyroid dysfunction, which greatly affect the quality of life (54–56). So, it is clear that over production of autoantibodies and its governance by regulatory T-cell (Tregs) and T-helper-17 cells (Th17) is prime in controlling immune-induced thyroid pathogenesis. The micro communications between T-cells and AITDs are mainly controlled by plasma microvesicles (57). Intercellular communication mediated by exosomes is helpful in regulating innate and adaptive immunity by delivering the target antigens, inflammatory factors presentation and killer proteins. Recent studies have reported that the crucial role of apoptosis in the maintenance of tissue homeostasis and the pathogenesis of immune diseases and AITDs were known for dysregulation of apoptotic signaling pathways (58). A study reported that excess iodide could induced apoptosis through TNF-related apoptosis-inducing ligand and death receptor expression (59), Iodinization increased TPOAb and TGAb, which lead to increased apoptosis *via* the Fas-mediated pathway (60). B cell-human lymphoblastoid cell lines derived exosomes enriched with Fas Lignad (FasL), which lead to activation of apoptosis in CD4+ T cells by Fas/FasL interaction, so exosomes can be a could be associated with cell apoptosis in Hashimoto’s thyroiditis (61). Immuno-modulatory actions and immune responses of cells are fine-tuned by exosomal surface protein markers like HSP60 and HSP70, which binds to various receptors like TLR, CD40, and Cd9 of the immune cells (62). However, specific mechanism of trans-communications and molecular switch at receptor level yet to be unraveled.

Graves’ disease is the most common cause of hyperthyroidism with manifestation of thyrotoxicosis and Graves’ ophthalmopathy and highest incidence in 30-50 aged women (63). An interesting studies by Rossi and others found that Dichlorodiphenlytrichloroetahns (DDT) stimulated thyroid follicular cells have active role in exosome mediated transferring the thyrotropin releasing hormones (TSHR) which involved in production of autoantibodies to the TSHR leading to Graves’ diseases (64). As mentioned before epigenetic modification (DNA methylation, histone modifications and noncoding RNAs(ncRNAs) interference) also plays a main role in pathogenesis of Graves’ diseases (65, 66), recent studies revealed that nucleic acids present in exosomes retain their pathological function in a recipient cell after direct transfer exosomal cargoes (67) and different stages of Graves’ diseases patients showed a different noncoding RNAs in exosome compared to healthy (68). Till now, exosomes related AITDs are still in sporadic stage. Till now, understanding of exosomes related AITDs are still in its infancy. Consequently, further research is required to elucidate the snapshot mechanism of exosomes in AITDS thereby delivers the deep understanding of AITDs, diagnosis and prognosis.

Drug induced liver injury (DILI) is common and almost all types of medicines. Recovery from DILI may require discontinuation medicines, hospitalization, or even liver transplantation (69, 70). Exosome are an attractive therapeutics and drug delivery vehicle, recently the hepatocyte and MSCs derived exosomes/EVs have revealed that they are effective in hepatic regeneration in liver injury models (71–73). Hepatocyte exosomes shown to have ceramidase and sphingosine kinase 2 (SK2) in their compartment, increased sphingosine-1-phosphate (S1P) in hepatocyte, which promotes proliferation hepatocytes (71). The MSC-EVs enriched in glutathione peroxidase 1 (GPX1) and knockdown of GPX1 reduced the protective effects of MSC-EVs in DILI model (74, 75). However, more preclinical experiments are still needed to understand roles of exosomes in DILI which can accelerate successful clinical translation of exosome therapy in DILI.

### 3.1 Exosomes in gut immune homeostasis

Gut cells are regularly exposed to foreign antigens, such as food molecules and bacteria. Partially digested and undigested food materials putrefy in the intestine through bacterial action, releasing several secondary metabolites of bacterial origin and further challenging the intestinal immune system. Inducing immune tolerance towards harmless food and bacterial antigens is essential to sustain progressive health devoid of routine inflammatory reactions. Defective immune tolerance can lead to inflammatory and autoimmune diseases.

**Figure 3:**
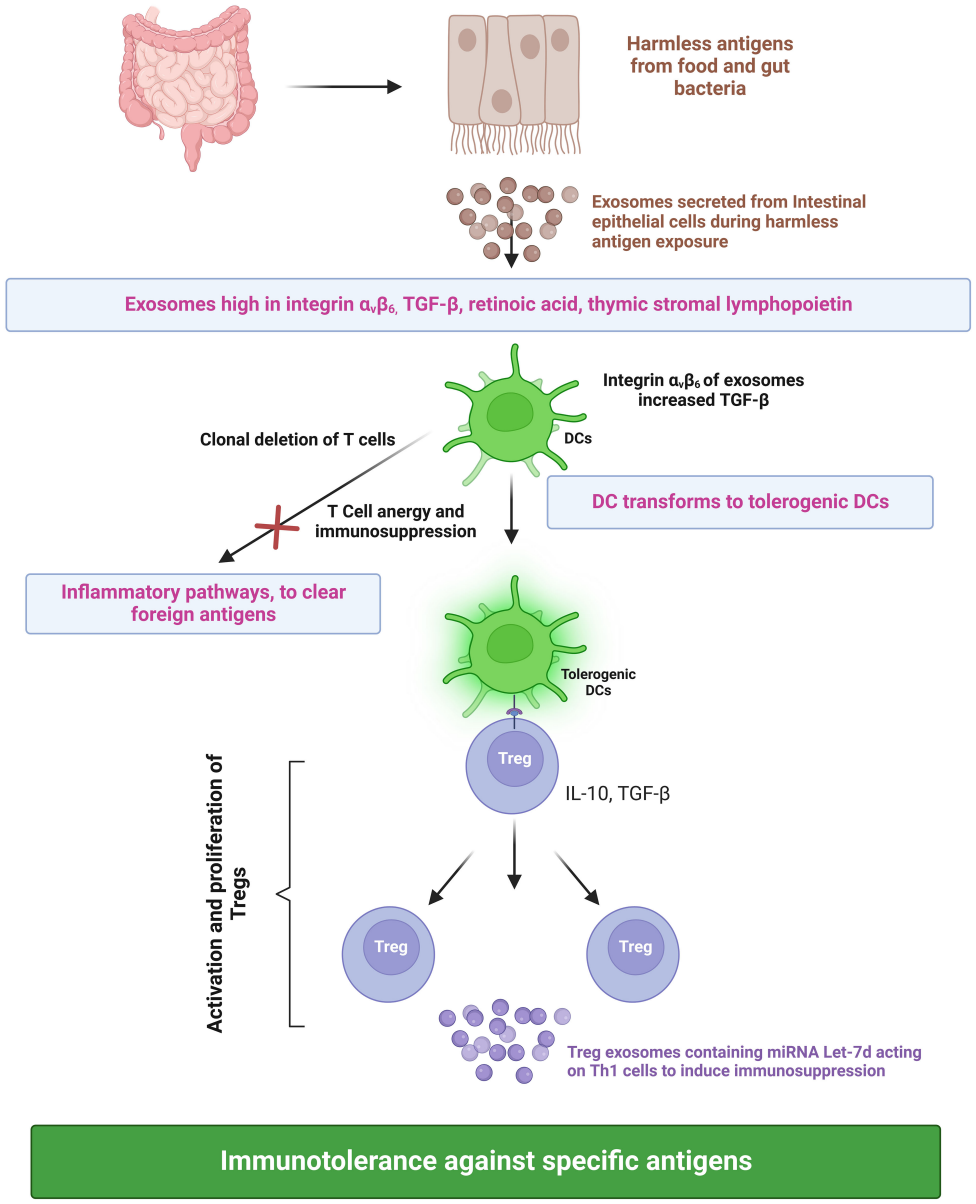
Roles of exosomes in inducing immune tolerance. TGF-b, Transforming growth factor beta; DC, Dendritic cell; IL-10, Interleukin 10; Treg, Regulatory T cells; Th1, T helper type 1; and miRNA, micro Ribonucleic Acid. Created with BioRender.com.

During infection, foreign antigens are presented to T cells by antigen-presenting cells. The dendritic cells play a pivotal role in controlling the effector and regulatory mechanisms of the immune response. A subset of dendritic cells called tolerogenic dendritic cells binds to T cells and suppresses the immune response against harmless food antigens or self-antigens, thus inducing immune tolerance (76). The presentation of antigens by tolerogenic dendritic cells results in the activation and proliferation of regulatory T cells (Treg), consequently leading to immune tolerance against a specific antigen (77). Furthermore, these semi-mature dendritic cells also induce clonal deletion of T cells and cause T cell anergy, resulting in an immunosuppression-dependent peripheral tolerance towards harmless antigens of the gut. Tregs execute their immunosuppressive functions by secreting cytokines (IL-10 and TGF-β) (**Figure 3**). Besides tolerogenic dendritic cells and Tregs, exosomes also play a vital role in immune response regulation. Exosomes secreted from intestinal epithelial cells (IECs) play a critical role in intestinal immune homeostasis regulation. IECs divert dendritic cells towards the immune tolerance pathway through TGF-β, retinoic acid, and thymic stromal lymphopoietin (IL-7 family). Exosomes secreted by IECs contain immunoregulatory molecules. During an infection, exosomes loaded with peptide-MHC II are secreted by IECs, taken up by antigen-presenting cells, and induce efficient T-cell activation. This would eventually lead to Th1- and Th17-mediated clearing of the foreign antigen/pathogen. IECs exposed to harmless antigens have an entirely different activation pathway that ensures the suppression of inflammation against harmless antigens. *In vitro* cell culture experiments simulating digestion using cells exposed to ovalbumin and digestive enzymes indicated that IECs have an increase in integrin αvβ6 expression with a corresponding increase in integrin αvβ6 in exosomes when exposed to harmless antigens. The uptake of integrin-loaded exosomes by dendritic cells increases their TGF-β expression and transformation into tolerogenic dendritic cells. This, along with T regulatory cell activation, results in the suppression of the immune response against harmless antigens (78). Additionally, Tregs secrete exosomes that elicit immunosuppressive activity (**Figure 3**) through the transfer of miRNA Let-7d to Th1 cells (79).

IECs secrete immunomodulatory exosomes, which have increased expression of MHC class II and Fas ligands, into the mesenteric lymph after trauma/hemorrhagic shock. This study also demonstrated that exosomes released after trauma/hemorrhagic shock significantly suppressed lipopolysaccharide-mediated CD80 and CD86 expression on dendritic cells and decreased their antigen-presenting capacity to induce lymphocyte proliferation (80). Exosomes secreted by the intestine after intestinal ischemia/reperfusion activate microglia, increasing the neuronal apoptotic rate and decreasing synaptic stability, thus leading to memory impairment (81). Exosomes produced by gut tropic T-cells regulate T-cell homing to the gut. These exosomes upregulate integrin α4β7 binding to mucosal address in cell adhesion molecule 1 (MAdCAM-1) expressed on endothelial venules in the gut, suppressing MAdCAM-1 expression in the small intestine, thereby inhibiting T cell homing to the gut (82). Human breast milk produces exosomes that decrease inflammation caused by necrotizing enterocolitis (83). Exosomes engage in activating the neuronal cells when intestinal cells are treated and activated with GABA (Gamma-aminobutyric acid). MicroRNAs in exosomes are responsible for this activation (84). These data collectively indicate a crucial role played by exosomes in the gut and gut-brain axis.

Exosomes produced by various cells are also involved in healing various gut-related diseases, such as inflammatory bowel disease (IBD), colorectal cancer, and intestinal barrier dysfunction. Proteasome subunit alpha type 7 was abnormally expressed in salivary exosomes of IBD patients (85). Moreover, exosomes extracted from the saliva of IBD patients contained approximately 2000 proteins, which were significantly altered compared with that in healthy individuals. Further research will reveal the importance of this variation in IBD pathology. Exosomes produced from *Curcuma longa* have the potential to inactivate the nuclear NF-κB pathway to ameliorate colitis and promote intestinal wound repair, which in turn alleviates IBD (86). Exosome-like nanoparticles obtained from grapes help induce intestinal stem cells and protect against dextran sulfate sodium (DSS)-induced colitis (87). Another study reported that oxaliplatin resistance in colorectal cancer was reduced with exosomes delivering miR-128-3p, consequently increasing the chemosensitivity of these cells (88). Mesenchymal stem cell (MSC)-derived exosomal miR-34a/c-5p and miR-29b-3p reportedly improve intestinal damage by targeting the Snail/Claudin signaling pathway. These exosomes increase the expression of Claudin-3, Claudin-2, and ZO-1 (89) (**Table 2**).

**Table 2:** Contents and functions of the exosome.

Sources	Major contents	Effects	References
Intestinal epithelial cells	MHC-II, Fas ligand	Apoptosis of dendritic cells.	(76)
Intestinal epithelial cells	MHC-II, Fas ligand	Microglial activation, a decline in synaptic stability, neuronal apoptosis, and cognitive impairment.	(77)
Human breast milk	Oligosaccharides, such as 2’-fucosyllactose and 3’-sialyllactose (3’-SL)	Decreased inflammation in intestinal cells.	(79, 90)
Treg cells	Let-7d	Th1 cell proliferation suppression.	(90)
Intestinal epithelial cells	integrin αvβ6	TGF-β upregulation in dendritic cells.	(77)
NSC-34 motor neurons	miR-124	Reduced phagocytic abilities and induced senescence in microglia cells.	(91)
Lymphocytes	miR-142-3p, miR-142-5p, and miR-155	Apoptosis of insulin-secreting beta cells.	(92)
Serum of patients with polycystic ovary syndrome	miR-424-5p	Inhibited granulosa cell proliferation and induced cell senescence.	(93)
Dendritic cells	MHC-I and MHC-II	T-cell-dependent anti-tumor response.	(4)

### 3.2 Asthma and exosomes

Asthma is a non-contagious disease that can develop owing to allergies. It is characterized by bronchial hyperresponsiveness, mucosal edema, and airflow restriction (94). Asthma progression requires interaction between resident and inflammatory cells (95). Exosomes released from eosinophils and innate immune cells are critical in regulating and enhancing asthma pathophysiology. Pathological changes in asthma are caused by the activation of structural lung cells and airway remodeling, which contain enzymes such as eosinophil cationic protein, eosinophil peroxidase, or major basic proteins (96) that cause epithelial damage (a hallmark of asthma). Apart from this, the exosomes also contain molecules such as nitric oxide (NO), lipid mediators, and ROS, which are responsible for inflammation (97). Previous reports confirm (97, 98) the abundance of exosomes containing these enzymes in individuals with asthma than in healthy individuals.

T lymphocytes, key players in the inflammatory response in asthma, are also known to produce exosomes. The exosomes produced by T cells activate and degranulate mast cells and release cytokines, leading to tissue remodeling and airway hyper-responsiveness (99). Similarly, exosomes released by B lymphocytes transport specific molecules of antigen-presenting cells, enabling these exosomes to present the antigen to induce T-cell responses and release IL-5 and IL-13 cytokines (100).

The expression of several proteins involved in allergic responses increases owing to the transfer of exosomes from airway epithelial cells to human tracheobronchial cells, which causes aggravation of asthmatic symptoms. Extracellular vesicles aid in disease progression by promoting dendritic cell maturation and increasing T helper cell 2 (Th2) proliferation (101). MSC-derived exosomes have disease-alleviating effects, such as decreased production of Type-2 innate lymphoid cells, Th2 cytokines, and mucus. In contrast, exosomes derived from bronchoalveolar lavage fluid inhibit specific immunoglobulins such as IgE and IgG1 (102).

Another study (103) suggested that asthma induction enhanced the levels of CD63 and acetylcholine esterase activity (exosome-associated enzyme), indicating an increase in exosome biogenesis and its secretory pathways. In asthmatic tissue samples, IL-4 levels were increased, triggering the central pro-inflammatory responses that regulate eosinophil transendothelial migration and IgE production. This can further increase mucus secretion and also accelerate Th2 differentiation. In contrast, IL-10 levels were decreased, which inhibited Treg activity (104). TNF-α, a pro-inflammatory cytokine, was also increased in the tissue samples. This correlates with an increase in exosome secretion and its participation in inflammatory pathways that function in asthma (100).

### 3.3 Arthritis and exosomes

Arthritis is the inflammation of the joints caused mainly by immune system dysfunction. It is an umbrella term for several diseases, such as rheumatoid arthritis and osteoarthritis. Osteoarthritis is a degenerative disease that damages the synovial joints of the hands, feet, knees, and hips, which in many instances, are disabled (105–108). The different tissues around the joints have various effects on the pathology of osteoarthritis, especially inflammatory cytokines from the synovial fibroblasts of inflammatory cells. IL-1β, a pro-inflammatory mediator, and bone-regulatory factors, including BMP-2 (bone morphogenetic protein-2) (67), promote articular cartilage damage and hasten osteoarthritis development by facilitating the release of various proteolytic enzymes (109).

Bone homeostasis is balanced by resorption and formation, and exosomes produced by osteoblasts regulate this process. These exosomes contain osteogenic signals, such as molecules of the eukaryotic initiation factor-2 pathway, which help bone formation (110). Exosomes produced from specialized cells, such as mineralized osteoblasts, promote the differentiation of bone marrow stromal cells into osteoblasts (111). MSC-derived exosomes also help maintain bone homeostasis by preventing cells from undergoing apoptosis during stress conditions, such as hypoxia and serum starvation (112). They also assist in healing fractures *via* the sprout-related EVH1 domain containing 1 (SPRED1)/Ras/Erk signaling pathway (113, 114).

IL-1β treatment reduced the expression of the anti-inflammatory gene TGF-β. Notably, exposure to exosomes derived from bone MSCs recovered TGFβ expression. This phenomenon was again confirmed by the observation that abnormally high expression of several pro-inflammatory genes, such as NF-κB and TNF-α, can be reduced by co-culture with exosomes (115). By exploiting this function of exosomes, their clinical applications for the treatment of bone disorders are being explored. As the cargo in the exosome is highly dependent on the release of host cells, changes, if any, in the tissue microenvironment are reflected in the exosomal cargo.

### 3.4 Psoriasis and exosomes

Psoriasis is a non-contagious, immune-mediated disease that causes scaly, raised patches on the skin owing to systemic inflammation. The immunopathology of psoriasis is characterized by an increase in the CD4+ and CD8+ T cells, neutrophils, natural killer cells, mast cells, and macrophages (116–118). Discoveries in this field have shed light on the increased numbers of IL-17-secreting T cells and elevated levels of the Th17-polarizing cytokine IL-23 in psoriatic lesions (119). Furthermore, small extracellular vesicles released from psoriatic keratinocytes transfer miR-381-3p to CD4+ T cells, which induces the polarization of Th1 and Th17 cells and eventually contributes to psoriasis development (120). Patients with generalized pustular psoriasis have a higher neutrophil-to-lymphocyte ratio than in healthy individuals. These neutrophils consequently induce a higher expression of inflammatory genes, including IL-1β, IL-18, and TNF-α. This occurs *via* the increased secretion of exosomes from neutrophils, which are then rapidly internalized by keratinocytes, thus increasing the expression of these inflammatory molecules *via* activating the NF-κB and MAPK signaling pathways (121).

Langerhans cells, prominently present in the epidermal layer of the skin, play a pivotal role in the pathogenesis of psoriasis. Unlike MHC receptors that present only peptides, the CD1a receptor of Langerhans cells presents a broad spectrum of lipid antigens to T-cells, which amplify inflammation in psoriasis mouse models (122). The T-cell-mediated inflammatory response in the skin is mediated by increased phospholipase A2 activity, and phospholipase A2 levels were reportedly high in patients with psoriasis. IFN-α increases the release of exosomes from mast cells. T cells associated with psoriasis identify exosomes carrying lipid antigens/phospholipases originating from mast cells. This can lead to increased IL-22 and IL-17A production by CD1a-autoreactive T cells (123). Neutrophils (NETs) released during NETosis are a major source of increased IL-17 levels in psoriasis (124, 125). TNF-α, IFN-γ, IL-2, IL-6, IL-8, IL-18, and IL-22 levels were higher in patients with psoriasis than in healthy individuals. However, this was not the case for IL-1β, IL-4, IL-10, IL-12, IL-17A, IL-21, and IL-23 (126). Another prominent feature of patients with psoriasis is that the levels of iron, hepcidin, and total iron-binding capacity of the exosomes were significantly lower. In contrast, the soluble transferrin receptor and heme oxygenase-1 levels were significantly overexpressed (127).

Luteolin could be used in treating psoriasis as it heals skin lesions and alleviates psoriatic symptoms by reducing the effects of IFN-γ, inhibits the expression and exosome secretion of HSP90, and modulates the proportion of T-cells in the plasma (128). Furthermore, topical application of MSC exosomes, known for their immunomodulatory properties, reduced IL-17 and terminal complement activation complex C5b-9 (129). Another study used MSC-derived exosomes to alleviate psoriasis-like skin inflammation *via* the IL-23/IL-17 axis. Exposure to exosomes reduces the levels of STAT3/p-STAT3, IL-17, IL-23, and CCL20, suggesting that exosomes are a potential therapeutic candidate (130).

### 3.5 Roles of exosomes in type-1 diabetes mellitus

Type-1 diabetes mellitus is an autoimmune disease characterized by the selective killing of pancreatic beta cells by the selective infiltration of T lymphocytes. Lymphocytes release exosomes containing miRNAs, which are involved in the differentiation and maturation of beta cells and regulate insulin release and survival. Exosomes released from pancreatic cells could be associated with cytokine stimulation and recreation, as exosomes contain diabetic autoantigens (92, 131). Exosomes released by adipose tissue can regulate gene expression, thus modulating interorgan communication (132). The various miRNAs in exosomes associated with diabetes mellitus include miR-142-3p, miR-142-5p, and miR-155 from lymphocytes that can cause selective death of insulin-secreting beta cells (133–135). Microarray evaluation revealed 25 miRNAs dysregulated in patients with diabetes, including miR-326, miR-186, miR-21, miR-126, miR-16, let-7a, let-7f, and let-7g, compared with that in the control. A correlation was seen connecting HbA1c, miR-326, and let-7a between plasma glucose levels and let-7f (132, 136). Individuals affected by type2 diabetes exhibit altered miR-20b-5p expression, which eventually leads to alterations in protein kinase-B (AKT) and glycogen synthase kinase (GSK), the main components of the insulin signaling pathway (137). Individuals with type 1 diabetes contained decreased miR-16-5p, miRNA-302d-3p, and miR-574-5p expressions, and increased miR-25-3p expression (138).

However, studies have reported that it does not affect the function of pancreatic glucagon-producing α-cells. The intracellular β-cell autoantigens in type 1 diabetes mellitus, namely, GAD65, IA-2, and proinsulin present in exosomes, are taken up by dendritic cells and, consequently, activated. Thus, the exosomal release of intracellular autoantigens and immunostimulatory chaperones induced by stress may initiate autoimmune responses in type1 diabetes mellitus (139). Pancreatic β-cells transfer secretory vesicles to phagocytic cells for presentation to T cells. The criterion for transfer requires the positioning of beta cells in close interaction with phagocytes under low-glucose conditions. This transfer is promoted by increased glucose concentration, as it increases cytosolic Ca^2+^ levels. Secretory vesicles from the pancreas transfer two sets of vesicles, one with insulin and another containing its catabolites (140), eventually facilitating their access to the immune system. Exosomes and exosome-like vesicles are used to diagnose and treat insulin resistance in type2 diabetes mellitus (141). Exosome-loaded immunomodulatory biomaterials have been used to alleviate the immune response in immunocompetent diabetic mice after islet xenotransplantation (141, 142). However, the comprehensive characterization of exosome-like vesicles is challenging, and their effects are not specific, which can lead to undesired consequences (141).

## 4 Roles of exosomes in organ transplant

For end-stage organ diseases, transplantation becomes the only therapeutic option left with patients (143). Regardless of advancement in the field of medicine and surgery, substantial portion of patients continue to suffer various post-transplant complications and rejection of transplants. In recent years, EVs (including exosomes) have fascinated boundless attention of the scientist and surgeons as exosomes becoming a potential tool for understanding the transplanted organ physiology and diseases. Numerous studies have shown that exosomes released by various antigen presenting cells, which involves in humoral and cellular immune system (144).

The transplanted organ releases exosomes (allograft-exo) with donor major histocompatibility complex (MHC) molecules and they go out from the graft to the recipient’s lymphoid system (145, 146). These allograft-exo are internalized by recipient antigen-presenting cells (APCs), which leads to presence of donor MHC molecules on recipient APCs’ surface. This is called as semi-direct pathways in T cell-mediated rejection without direct contact between donor cells with recipient APCs (146–148). In a skin graft mice model, the donor MHC molecules are transported by EVs to the host’s lymphoid organs, then dendritic cells/B cells take up this donor MHC molecules (MHC-I and MHC-II) and presented to T cells, which leads to a direct inflammatory alloresponse in mice (146, 149). In case of solid organ transplantation, for example the heart transplant, recipient’s blood vessels are connected to the transplant during surgery. This leading to donor passenger leukocytes from graft may move to recipient, reported that donor dendritic cells was present with in the spleen of allogeneic heart transplanted mice (150), this was further reported in other studies that allo-MHC cross-dressed cells in lymphoid organs *in vivo* after cardiac transplantation (151, 152), allograft-exo may have been involved as well. Some studies show that allogeneic exosomes under certain circumstances involved in tolerance rather than rejection of allografts (153, 154). However, exosome involvement in organ transplantation still has not been elucidated and extensively studies are needed.

## 5 Cross talk on exosomes and cell receptor

Inborn or innate immunity is inherited immunity consists of cellular and hormonal arms. The intercellular communication during the human genesis is first drafted by cell “language” communication system which is mediated by exosomes to maintain the rigidity of the cell. The effective immune response to foreign materials, like pathogen associated molecular patterns (PAMPS), danger associate molecular pattern (DAMPS), or life style associated molecular pattern (LAMPS), is controlled by exosome communication language. PAMPS and DAMPS are structurally diverse external molecules from pathogenic bacteria which includes, lipids, proteins, carbohydrates and material by products. Mega receptors in defense cell like leukocytes, first will have defense mechanism during the bacteria, virus, parasites and fungi invasion. Cellular recognition patterns such as Toll like receptors (TLRs) are expressed in various immune cells like leukocytes, macrophages, dendritic cells, B cells, T cells, and even non-immune cells like fibroblasts and epithelial cells (155). TLR first binds to PAMPS, and DAMPS thereby ignites the cell signaling cascades as combat mechanism. Under sterile inflammation condition, parenchymal and stromal cells communicate *via* extracellular vesicles through innate system. For example, during noxious gas inhalation, in lung, epithelial cell produces more exosomes with excess loading of microRNA17/221 (miRNA17/221) thereby promoting the local inflammation through the recruitment of M0 macrophage (156). Type 1 transmembrane protein, TLR has two domains like leucine-rich repeats (LRR) motif and Toll/LI-1R (TRR) as cytoplasmic domain in all immune cells and having different sub types like TLR-1, 2, 4, 5, 6 as cell surface members and TLR-3, 7, 8 and 9 as intracellular components (157). Among all these TLR-2 recognizes pathogens metabolites like lipoproteins/lipopeptides, peptidoglycan, glycosyphosphatidylinositol, phenol soluble modulin, zymosan and glycolipids (158). Double stranded RNA (dsRNA) of viruses will be recognized by TLR-3, while TLR-7 perceive GU rich single stranded RNA (ssRNA) but TLR-5 spots the bacterial flagellin (159–161). In tumor development and subsequent metastatic microenvironment, series of coordinated events are taking place between exosomes and TLR, thereby it plays an important role in the cancer development process. In cancer microenvironment scenario, TLR-8 plays important role by transmitting ECmiRNAs like miRNA 21 and miRNA 29a because of highest binding capacity with TLR-8 receptors thereby activates the receptors in immune cells and subsequently leads to the premetastatic inflammatory cytokines (162).

Despite the evidence that exosomes do play important role in the intercellular communications, the production and transport rely on the donor cells physiological conditions and receptor activations of receiver cells. TLR is known to be an importance factor in acute kidney diseases like urinary tract infections, ischemia-reperfusion (IR) injury, lupus nephritis and diabetic nephropathy (163). Micromanagement of exosomes in renal physiology have been studied both *in vitro* and *in vivo* condition. Miyazawa et al. first reported the role of exosome mediated aquaporin 1 and 2 (AQP-1, AQP-2) transport in collecting duct cells (164). Lipopolysaccharide (LPA) induced renal tubular epithelial cells showed the elevated level of exosome miRNA 19b-3p thereby induce the macrophage dependent tubulointerstitial inflammation (165). Extensive investigation on exosome mediated urolithiasis (kidney stone disease) remains under investigated, indeed, decreased the level of miRNA-21and Let-7 was noticed in lupus nephritis patients (166). However, genetic manipulations of specific transcriptomes aided with exosome engineering would shed better understanding of kidney stone disease (167).

## 6 Exosomes in hematological complications

Potential role of exosomes in immunomodulation disorder in blood cancer or the induction of hematological diseases in polycythemia vera (PV), essential thrombocythemia (ET) myelofibrosis (MF) (168). Chronic Myeloid leukemia (CML) cells (K562) derived exosomes promoted the angiogenesis, which was inhibited by dasatinib through inhibition of exosome release from K562 CML cells and their microenvironments (169). This *in vitro* study strongly supports hypothesis that microenvironment driven by K562 CML cells derived exosomes governs the clinical manifestation.

These exosomes also contains angiogeneic miRNAs such as mir-92 which helps in tumor cell migration during hypoxic condition (170). Disturbed HIF-1 pathways with upstream and downstream associated gene regulations by miR-1555, mir210, and mir135b also plays important role in migration of the CML cells by suppress or activation of factor inhibiting hypoxia inducible factor 1 (FIH-1). Taken altogether, cumulative factors such as EVs, growth factors cytokines and miRNAs play a fascinating role in the leukemogenesis, and cross-talks between various cell populations *via* each of the indicated components promote the formation of different types of hematological malignancies, including acute myeloid leukemia (AML), acute lymphoblastic leukemia (ALL), chronic lymphocytic leukemia (CLL), chronic myeloid leukemia (CML), lymphoma, and multiple myeloma (MM) (171).

## 7 Autophagy and exosomes relationship in cancer

The body uses autophagy to eliminate unhealthy cells so that it can replace them with new, healthier ones. In normal conditions, long-lived proteins and old organelles were degraded by autophagy for restoration of cellular contents. But under stressful conditions, for example hypoxic (lower oxygen) conditions, autophagy is activated to recycle molecules for providing energy and nutrients (172–174). Studies on exosomes have enlightened our understanding of their biological functions and recently several studies have shown that cancer exosomes play a great role in tumor progression and metastasis (175, 176). The studies have reported that knockdown of both autophagy related 16 Like 1 and autophagy related 5 in breast cancer cells shown to produce and release lesser exosome, this leads to decrease tumor metastasis (177, 178). Studies on G alpha interacting protein (GAIP) and GAIP interacting protein C-terminus have been shown involves in stimulation of biogenesis of exosome and autophagy flux in pancreatic tumor cells (179, 180). Inhibition of autophagy related 12–autophagy related 3, a complex plays important role in late step of autophagosome formation, reduce the exosome biogenesis by disrupting late endosome trafficking in multivesicular body (MVB) through interacting with Alix (172, 181). Several studies have well documented that autophagy, exosome plays important role in tumor progression (172, 182, 183). Autophagy and exosome release are robustly activated in tumors, which strongly showing that both these pathways are play a interplay in between and they are as part of hallmark of cancer cells.

Recently the scientists are focusing on inhibition of tumors progression and metastasis by targeting the autophagy and exosome biogenesis. A study reported that sulfisoxazole targeting the endothelin receptor A, which lead to inhibition of exosome biogenesis through increased degradation of MVB *via* the autophagy–lysosome pathway (184). A Study suggested that an inhibitor of ULKs (a serine/threonine protein kinase) leads to accumulation of immature early autophagosomal structures (185); ULK1 are involved in the trafficking of autophagy related 9, which is main player in intraluminal vesicle formation within amphisomes and autolysosomes (186, 187). Likewise other several inhibitors have been studied to inhibit biogenesis of exosomes (185, 188). Still several preclinical and clinical studies are required to understand the role of cancer exosome and autophagy and their targeting in cancer treatments.

## 8 Exosomes in diagnostics

Exosomes are released by cells, and their components provide a brief story about the microenvironment in and around the cell. These could be signals, exosomes presenting antigens, or even cancer-promoting factors. This property has previously been exploited for diagnostic purposes. The increased and highly stable exosome expression in patients with cancer renders it an up-and-coming player for cancer diagnosis. Various groups have used exosomes present in the plasma for diagnostic purposes. Different immunological markers of exosomes have been characterized in patients with chronic lung allograft dysfunction. Lung transplant recipients who displayed both the phenotypes of obstructive bronchiolitis obliterans syndrome and restrictive allograft syndrome had exosomes with distinct molecular and immunological profiles. Upon further testing, restrictive allograft syndrome samples were observed to have a higher concentration of pro-inflammatory factors, indicating severe allograft injury (189). Exosomes have emerged as prominent biomarkers for cancer diagnoses. Another group investigated the prominence of plasma-derived exosomal miR-19b in the diagnosis of pancreatic cancer. The results suggested that the levels of Exo-miR-19b normalized using miR-1228 were comparatively lower in patients with pancreatic cancer than in healthy individuals. This indicates that exosomes are promising candidates as biomarkers (190). This property of exosomes is utilized to diagnose various types of cancers, such as non-small cell lung cancer (NSCLC), prostate cancer, osteosarcoma, and cervical cancer. Lower expression of circulating miR-651 was observed in patients with cancer than in healthy individuals. Moreover, exosomes collected from HeLa cells were rich in CD63, CD9, and CD81 proteins, which are cancer cell hallmarks (191).

Long non-coding RNA (lncRNA) expression in urinary exosomes of NSCLC patients and healthy individuals as a potential lung cancer diagnosis has been explored. The results indicated that differential lncRNAs in urinary exosomes are NSCLC biomarkers because lncRNAs are enriched in specific pathways that might be involved in tumor cell proliferation and other processes associated with NSCLC pathogenesis (192). Liquid biopsy of exosomes isolated from patients with prostate cancer revealed that exosomes are enriched for genes that are hallmarks of prostate cancer, such as androgen receptor, kallikreins (KLK2), cyclin-dependent kinase inhibitor 1A (CDKN1A), KLK10, JUN, and B2M (β_2_ microglobulin). These observations indicate that exosomes transport critical disease RNA transcripts and can be used as non-invasive diagnostic biomarkers (193). Exosomes of patients with prostate cancer had higher amounts of survivin, an apoptosis inhibitor, which is another biomarker for early detection of prostate cancer (194). These examples suggest that this is an untapped niche for early cancer diagnosis and has potential.

One of the first examples of exosome biodistribution in the body by *in vivo* positron emission tomography (PET) for non-invasive monitoring of copper-64 (64Cu)-radiolabeled polyethylene glycol (PEG)-modified exosomes was demonstrated with high imaging quality and quantitative measurements of blood residence and tumor retention. A fluorescent dye, amine-reactive Alexa Fluor 488 (NHS-Alexa Fluor 488), was conjugated to exosomes to form Alexa Fluor 488−exosome and Alexa Fluor 488−exosome−PEG. PEGylation confers an enhanced pharmacokinetic profile and higher tumor accumulation in exosomes compared with that of native exosomes. However, it also reduces premature hepatic sequestration and clearance of exosomes, thereby highlighting their improved therapeutic delivery efficacy and safety (195). Notably, this study provides crucial guidelines for obtaining precise and quantitative information on the biodistribution of exosomes *via* surface engineering, radiochemistry, and molecular imaging, which may aid future exosome research.

A technique was devised for *in vivo* neuroimaging and exosome tracking using gold nanoparticle labeling (196). Exosomes may be directly labeled with glucose-coated nanoparticles (GNP) without the need to label parent cells, and this labeling occurs through an active mechanism linked to the GLUT-1 (glucose transporter-1). Intranasal delivery is more effective than intravenous injection for brain accumulation. The noninvasive intranasal delivery route has multiple benefits and is a potential and effective treatment option for various CNS disorders. In contrast to the non-lesioned brain, the detection and accumulation of MSC-derived, GNP-labeled exosome labeling in the stroke region of the brain up to 24 h after administration was observed. Multifunctional exosomes for cancer theranostics were developed by electroporating urinary exosomes and ultra-small gold nanoparticles with Ce6 (chlorin e6) to obtain real-time fluorescence imaging and improve photodynamic therapy (197). The nanocomposites cloaked with exosomes exhibited enhanced long-term retention, biocompatibility, and penetrating behavior than that of free Ce6. A comprehensive study of the theranostic application of exosomes in cancer has been described by Ailuno et al. (198).

Circulating tumor exosomes with specific biomolecules have been recently used for detecting cancers and assessing therapy response and they are becoming more common as diagnostic targets (199–201). In recent times an ExoChip (with an anti-CD63 antibody), microfluidics device constructed to capture and collect specific exosomes, it showed that exosomes were significantly higher from individuals with cancerous diseases than healthy controls (202). Liquid biopsy with exosomal IGF-1R expression is used in individuals with lung cancers instead of invasive traditional tissue biopsies, which was achieved by microfluidics exosome analysis platform (203). A microfluidics chip is used for evaluation of circulating EpCAM-positive exosomes with plasma exosomes. EpCAM-positive exosomes was higher in individuals with breast cancer than healthy individuals (204). These studies and several other studies suggest that microfluidics-based exosome isolation and detection methods are more reliable (205) but still further discoveries are needed for its routine clinical translation.

## 9 Exosomes in therapeutics

The role of exosomes for therapeutic purposes requires further elucidation; nonetheless, their potential has been well-established. Exosomes did not exhibit toxicity upon injection, and nano-sized membrane-bound vesicles were well tolerated by the body. These properties make them excellent candidates for contact delivery because they do not undergo degradation. Both targeted and non-targeted exosome deliveries successfully alter protein expression in cancer cells (206).

There are several methods for isolating and purifying exosomes. Based on yield and purity, acoustic nanofilter (207), ExoSearch chip (208), immunoprecipitation (209), density gradient (210), precipitation kits, and ultracentrifugation methods are in use. A recent study demonstrated a separation method based on acoustofluidics by integrating acoustics and microfluidics approaches to isolate exosomes from blood directly [140]. This approach combines cell removal with exosome isolation. By integrating these modules into a single chip, exosomes were isolated with a blood cell removal rate of over 99.999% (211). Another study described a novel approach using microfluidic devices to isolate exosomes from whole blood, directly facilitating translation to the clinic (212). The choice of the device has a significant impact on the yield and purity of exosomes in the case of cell lines and complex biological fluids. The isolation of exosomes can be validated using nanosight tracking analysis, transmission electron microscopy, or western blotting. The revolutionized development of different approaches for exosome isolation and purification has made procedures more feasible for early diagnosis and therapeutics.

The off-target binding of drugs leads to several adverse effects (213). Several research groups are investigating targeted drug delivery to obtain high efficacy and low toxicity. Exosomes play a significant role in the targeted delivery of drugs and drug-like molecules. Similarly, paclitaxel (PTX)-loaded AS1411-chol exosomes (AS1411: a nucleolin-targeting aptamer) were delivered to target cancer cells with high efficiency in a recent study (214). Surface engineering, genetic engineering, chemical modification, and membrane fusion are some approaches adopted for the targeted delivery of exosomes (12). By using targeted surface engineering delivery and increasing exosome concentration at disease sites, this approach can reduce toxicity and increase therapeutic efficacy (215). In genetic engineering, ligands or homing peptides can be fused with TMP (5,10,15,20-tetrakis (1-methyl-4-pyridinio)), which is expressed on the surface of exosomes (216). In chemical modification, any functional group can be modified by another; for example, alkynes can be modified by an amine group. The exosomal lipid bilayer membrane can spontaneously fuse with other membranes using a membrane fusion approach.

Targeting exosomes to deliver chemotherapeutics, such as doxorubicin, to breast cancer tumor tissues in mice has been evaluated. The results were substantial as the treatment caused enhanced and rapid tumor regression without toxicity than that with standard therapy (217). Another study successfully used exosomes as a delivery vector to transport PLK-1 siRNA to bladder cancer cells *in vitro*, resulting in selective gene silencing of PLK-1 (218). ACTN4 is highly expressed in exosomes from patients with castration-resistant prostate cancer (CRPC). RNA interference-mediated ACTN4 downregulation significantly attenuates cell proliferation and tumor invasion, thereby confirming its role in prostate cancer development (219). Exosomes produced by adipose-derived stromal cells (ASCs) can also be used in prostate cancer therapy. ASC-derived exosomal miR-145 could reduce Bcl-xL activity and promote prostate cancer cell apoptosis *via* the caspase-3/7 pathway (220).

Phosphatidylserine (PS) expressed on the surface of exosomes is linked to T-cell immune suppression. Recently, a novel PS-binding molecule was developed that successfully blocked the immunosuppressive activity of human ovarian tumors and melanoma-associated exosomes. Upon treatment with ExoBlock, T-cell-mediated tumor suppression was significantly enhanced along with an increase in the number and function of CD4 and CD8 T-cells, which are involved in reducing tumor metastasis, thus providing promising antitumor therapy (221). Exosomes isolated from human umbilical cord MSCs were transfected with miR-3182. Exosomal miR-3182 significantly reduced cell migration and proliferation in triple-negative breast cancer (TNBC) cells. We also observed that miR-3182 loaded exosomes induced apoptosis in TNBC cells by downregulating mTOR and S6KB1 expression. Thus, this treatment can reduce the invasiveness of TNBC cells (222). Exosome therapy has also been used to treat other diseases.

In psoriasis, phospholipases on exosomes are associated with disease progression. Inhibition of phospholipases suppresses psoriasis progression (223). Inflammatory cytokines in the IL-23/Th17 axis and TNF-α signaling can be targeted. However, these can cause toxic side effects over an extended period (224). Alternatively, exosomes derived from MSCs have been used as potential treatments. This was tested in a mouse skin psoriasis model. The levels of pathology-associated IL-17, IL-23, terminal complement complex, and C5b-9 complex were reduced in the skin of mice treated with exosomes relative to that in the control (129).

Exosomes derived from adipose-derived MSCs (ADSCs) have therapeutic applications in hepatic I/R injury. ADSC-derived exosomes improved liver function by maintaining mitochondrial homeostasis through mitochondrial fission inhibition and promoting mitochondrial fusion and biogenesis. This may be attributed to the exosome-induced increase in the expression of mitochondrial fusion proteins such as OPA-1, MFN-1, and MFN-2. In contrast, they decreased DRP-1 and Fis-1 mRNA and protein expression related to mitochondrial fission. Additionally, exosomes significantly increase the expression of PGC-1α, NRF-1, and TFAM genes and proteins related to mitochondrial biogenesis (225). Another study in hindlimb ischemic animal models reported that MSC-derived extracellular vesicles activated VEGF receptors in endothelial cells, increased neovascularization at the site of ischemia, and accelerated recovery (226). In an associated study, extracellular vesicles derived from RAW 264.7, macrophages could also induce angiogenesis *in vitro* and *in vivo* (227). Exosomes derived from MSCs can target endogenous MSCs to enhance osteogenic differentiation and reduce adipogenic differentiation, which is a promising therapy for osteoporosis (228). Other possible treatment methods could be effective in initiating bone repair, regulating the immune response, and preventing bone resorption. Similarly, MSC-derived exosomes have also been used to treat inflammatory disorders, such as neurological disorders. This is accomplished using mRNAs, miRNAs, and immunosuppressive factors sourced from MSCs (229). Exosomes produced by MSCs stimulated by IFNγ reduced demyelination, decreased neuroinflammation, and upregulated the number of CD4, CD25, FOXP3, and regulatory T cells within the spinal cord. Moreover, exosomes reduce the levels of pro-inflammatory Th1 and Th17 cytokines; hence, exosomes can also serve as therapeutic targets for neurodegenerative disorders (230). Another promising study in this direction is from the Shetty group, which demonstrated that extracellular vesicles isolated from induced pluripotent stem cells effectively induce neurogenesis and treat neurodegenerative disorders in animal models (231). The hallmark of patients with pulmonary embolism is that pulmonary epithelial cells (PECs) are resistant to apoptosis. Mao et al. used exosomal miR-28-3p, which upregulated miR-28-3p expression and increased apoptosis by targeting apoptosis inhibitor 5 (API5) in PECs (229).

Milk-derived exosomes (mExosomes) are one of the most economical and promising drug delivery systems. Milk, an easily accessible raw material, can be prepared in substantially large amounts. Uniform particle size of ~100 nm, unique phospholipid layer (232), low immunogenicity, and inflammatory response (233, 234) make mExosomes an attractive research topic and an ideal vehicle for drug delivery in the future. The anticancer properties of drug-encapsulated mExosomes demonstrated *in vitro* growth inhibitory action in breast (T47D and MDA-MB-231) and human lung (A549 and H 1299) cancer cell lines (235). Animal studies on anti-tumor activity in mice bearing xenografts of A549 lung tumors exhibited low cytotoxicity and high bioavailability of mExosomes in the organs of the experimental model. A similar study reported that curcumin-loaded mExosomes exhibit enhanced anti-inflammatory and antitumor activities. This study also revealed that curcumin-loaded mExosomes have higher bioavailability than free curcumin (236).

RNA-based therapy has recently gained attention, but the challenge involves the delivery of highly unstable RNA molecules to biological systems. Gene expression studies have demonstrated the compatibility of mExosomes in delivering miR-148a-3p to hepatic (HepG2) and intestinal (Caco-2) cell lines (236), suggesting that mExosomes can be utilized as carriers of functional microRNAs. In a recent study (237), the mucus penetrability of siRNA-loaded exosomes was enhanced by adding a hydrophilic surface coating of PEG. PEGylated mExosomes exhibit improved penetrability and stability in an acidic gut environment. Bovine mExosomes are reportedly considered therapeutic agents against arthritis. Studies have demonstrated that spontaneous polyarthritis in IL-1Ra-deficient mice and collagen-induced arthritis is diminished by oral administration of mExosomes (238). However, the mechanism underlying the delayed disease onset remains unknown. Bovine mExosomes containing immunoregulatory miRNAs and anti-inflammatory proteins might have targeted inflammatory pathways. These are just a few examples of using exosomes for therapeutic purposes; further discoveries and breakthroughs have yet to be made.

## 10 Conclusion

Research on exosomes suggest that they are representative of the cellular condition and play a crucial role in upregulating, stabilizing, or down-regulating that condition. Exosomes secreted from a diseased cell would support disease progression, such as promoting angiogenesis for cancer or enhancing receptor expression to support viral infection or enhance inflammation. Exosomes secreted from stem cells reflect their purpose of supporting regeneration, healing, and growth. This information indicates that exosomes could be the next-generation technology that could receive large-scale public acceptance owing to their success in non-invasive diagnostics and non-toxic, target-specific drug delivery techniques used in clinical trials.

## Author contributions

PG, HM and B-CA contributed to the conception, writing, and discussion of this manuscript. All authors wrote the initial draft of the manuscript. All authors Contributed to the critical conclusion of the manuscript. All authors contributed to the article and approved the submitted version.
